# The German cochlear implant registry: one year experience and first results on demographic data

**DOI:** 10.1007/s00405-024-08775-x

**Published:** 2024-07-08

**Authors:** T. Stöver, S. K. Plontke, W. K. Lai, T. Zahnert, O. Guntinas-Lichius, H-J. Welkoborsky, A. Aschendorff, T. Deitmer, A. Loth, S. Lang, S. Dazert

**Affiliations:** 1 Department of Otorhinolaryngology, Goethe University Frankfurt, University Hospital, Frankfurt, Germany; 2 Department of Otorhinolaryngology, Head and Neck Surgery, Martin Luther University Halle- Wittenberg, Halle (Saale), Germany; 3 INNOFORCE Est, Ruggell, Liechtenstein; 4 Department of Otorhinolaryngology, Dresden University Hospital, Technical University, Dresden, Germany; 5 Department of Otorhinolaryngology, Jena University Hospital, Jena, Germany; 6 Department of Otorhinolaryngology, Nordstadt Hospital, Hannover, Germany; 7 Department of Otorhinolaryngology, Freiburg University Hospital, Freiburg im Breisgau, Germany; 8 Department of Otorhinolaryngology, Essen University Hospital, Essen, Germany; 9 Department of Otorhinolaryngology, University Hospital (St. Elisabeth Hospital), Bochum, Germany; 10 General Secretary, President or Past-President of the German Society for Otorhinolaryngology, Head and Neck Surgery e.V., Head Office, Bonn, Germany

**Keywords:** Registry, Cochlear implant, CI, Guideline, Whitepaper, Certification, Quality control, Health services research

## Abstract

**Purpose:**

Clinical registries have great potential for quality control of medical procedures regarding the indications, therapeutic processes and results, including their possible complications. This is particularly true when providing patients with severe hearing loss or deafness with a cochlear implant (CI). This treatment represents a lifelong care process that requires continuous quality control over time. On the initiative of the Executive Committee of the German Society of Otorhinolaryngology (Deutsche Gesellschaft für Hals-Nasen-Ohren-Heilkunde, Kopf- und Hals-Chirurgie e.V., DGHNO-KHC), a national German CI registry (Deutsches Cochlear Implant Register, DCIR) was established in January 2022. This article focuses on the first demographic and baseline data of the DCIR.

**Methods:**

The DCIR covers the complete therapeutic process from indication, surgery, fitting and lifelong aftercare in CI therapy. By the end of 2022, 75 hospitals in Germany had agreed to contribute to the DCIR.

**Results:**

During the year 2022, 63 hospitals actively contributed data to the DCIR. Pseudonymized data from 2,292 CI implantations (2,176 primary implantations, 99 explantations with immediate re-implantations and 17 re-implantations following an earlier explantation) in 2,108 patients were documented. Cochlear implantation was accomplished in 1,807 adults (≥ 18 years) and 301 children (< 18 years). Fourty patients (1,9%) were children < 1 year of age and 55 (2,6%) were patients > 85 years. From the total of 2,292 implantations, 226 (9.9%) were performed as simultaneous bilateral implantations (CI implantation in both ears of 113 patients on the same day of surgery) and 412 implantations (19.1% of 2,162 implantations with data provided on the contralateral ear’s hearing status) were in patients with single sided deafness (normal hearing in the contralateral ear). In addition, the reported complications in 2022 were also evaluated. Seven reports (0.4%) of mild to moderate severe facial nerve dysfunctions were documented. No reports of severe or total facial nerve dysfunction (House-Brackmann grade V/VI), meningitis or death related to CI therapy were documented.

**Conclusion:**

Although still in the start-up phase, these initial DCIR data already provide an interesting first insight into the demographic structure and baseline data of CI therapy in Germany. The successful implementation of the DCIR represents an important step towards continuous quality control of CI care.

## Introduction

Cochlear implant (CI) surgery is considered to be the standard of care in hearing rehabilitation for patients with profound hearing loss or deafness [1]. This method was continuously improved over the past decades resulting in significant changes in the indications, the surgical procedures and the entire care process. In particular, the long-term follow-up of treated patients provides important information concerning the clinical results achieved as well as potential complications. Within this context, medical registries provide an important contribution by creating a transparent and scientific database that is based on large numbers of cases, long observation periods and real-life data. Medical CI registries have already been successfully implemented in several countries worldwide. In 2021, 4 out of the 42 European countries had already successfully implemented a CI registry [2]. The Swiss and French CI registries, which are already contributing towards the quality control of CI care in their respective countries, should be mentioned here as examples [3, 4].

The latest medical standards for each country are usually defined as Clinical Practice Guidelines (CPG). In Germany, the most recent version of the CI-CPG was issued in 2021. This document represents a milestone for the long-term quality control of CI care in Germany, as it includes elements of structural, process and outcome-relevant quality for the first time [5]. For instance, this guideline specifies the number and qualification of persons involved in the process (e.g. qualification of the CI-Audiologist) as well as the entire care process, including lifelong aftercare [5]. Based on this guideline, the German Society for Otorhinolaryngology, Head and Neck Surgery (DGHNO-KHC) developed a “White Paper on Cochlear Implant care in Germany”, which corresponds to a practical recommendation for the implementation of the CPG content [6]. These documents provided the required foundation for the implementation of a medical registry for long-term collection of data on CI care in Germany. The basic structure of the “German Cochlear Implant Register” (DCIR) has been published recently [7].

After a preparatory period of several years, the DCIR began its active operation in 2022. The registry is scientifically supervised by the Executive Committee of the DGHNO-KHC and technically developed and implemented by a commercial registry operator (INNOFORCE Est., Liechtenstein) [8].

The registry provides documentation on the entire CI care process, which covers the indication, surgery, basic therapy (fitting), follow-up therapy (rehabilitation) and lifelong aftercare. Data collection is pseudonymized. Participation in the registry is voluntary for ENT hospitals. Data analysis is conducted with anonymous data provided by the registry operator. The aim of this study was to present initial results after one year of operation and actively enrolling patients to illustrate possibilities, as well as challenges, of implementing a national registry. The analysis presented here will focus on the demographic and baseline data.

## Materials and methods

### Infrastructure of the CI-registry

In the initial stage of conceptualizing the DCIR, a notable diversity became apparent in the existing databases and documentation systems across various hospitals for ensuring the quality of CI care. It was therefore essential to design technical solutions that could accommodate the various starting points of all participating hospitals, while guaranteeing uniform and high data quality within the DCIR. The executive board of the DGHNO-KHC thus decided to implement the DCIR in collaboration with a professional registry operator (INNOFORCE Est., Ruggell, Liechtenstein) [9], specialized in medical database systems for otorhinolaryngology.

Three technical avenues for data transmission were developed. They included:

A) use of a newly created web-portal supporting browser-based data entry [10].
B) implementing an individualized Application Programming Interface (API) that communicates with the existing local customized databases in the participating institution, or.
C) implementation of a special ENT database (ENTstatistics, INNOFORCE Est., Ruggell, Liechtenstein) from the registry operator [10].

In 2022, 63 out of 75 hospitals were technically able to provide data to the DCIR. Data transfer through the web-portal was used by 51 hospitals, while 12 hospitals chose to use ENTstatistics. None of the hospitals chose to transfer data via an individualized API data interface from an existing local custom database.

### Clinical data

The registry is designed to collect prospective data from CI surgeries (implantations and explantations). Details about the CI surgery data collected within the DCIR have been published previously [9]. In brief, data regarding clinical baseline data, preoperative audiometry, preoperative hearing history, implant, surgery, CI-related complications, CI use and rehabilitation progress, postoperative audiometry, hearing/language development (for children), and quality of life are included. The registry also documents details on the CI manufacturers and on CI-related adverse events. Yearly outcomes on hearing rehabilitation and audiometric measures are documented.

### Data protection, informed consent, and annual reports

The acquisition of clinical data for the DCIR requires individual patient consent, even though the data are fully pseudonymized when transferred to the registry from the hospital. Consequently, each participating institution sought consent from every patient whose data was transferred to the DCIR. For this purpose, a standardized consent form was developed. Identification of individual patient data or other data is impossible for both the registry operator and the executive committee of the DGHNO-KHC post-transfer to the DCIR. To maintain confidentiality, each participating hospital receives their own data as an anonymized annual report. The anonymized institution-specific data, such as complications, were compared against the national aggregated data of the DCIR. This allowed a benchmark comparison for each hospital on an anonymized basis. In addition, the executive committee of the DGHNO-KHC receives an anonymized annual national report.

The work presented here involved the analysis of only anonymized registry data and a retrospective data evaluation with the aim of quality assurance. Ethical approval was granted from a local ethical committee (2024 − 1662).

### Data analysis

The data analysis presented here is based on the first 12 months of the registry (January to December 2022, with data extracted on January 21, 2024). The data report was automatically generated by the registry operator. The containing anonymous data were then reviewed for plausibility and created the basis for this manuscript.

## Results

### Demographics

**Fig. 1:**
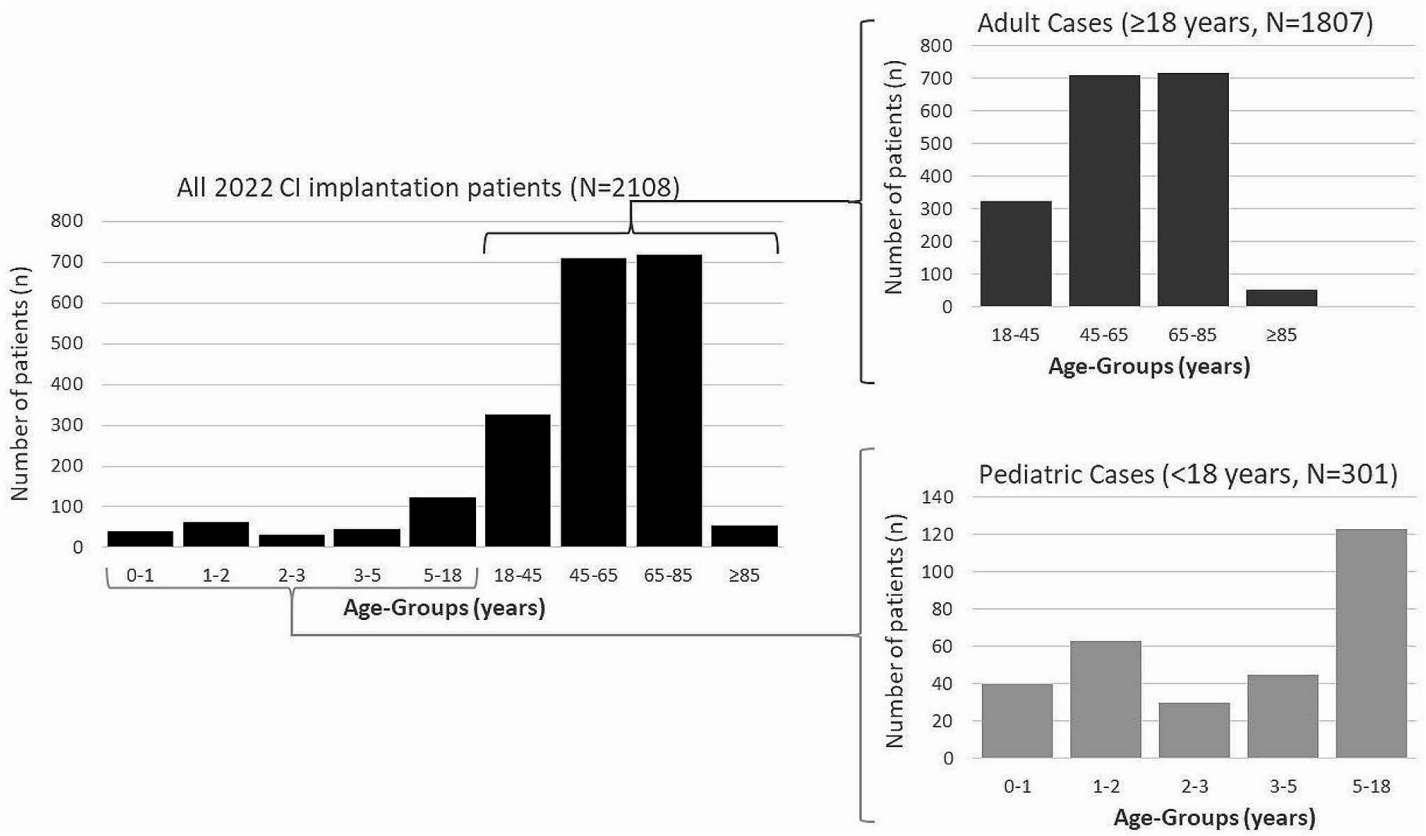
Age distribution of the 2108 patients who received a CI in 2022. Right: detailed distribution of adult cases (upper panel) and pediatric cases (lower panel)

In 2022, data from 2,296 CI surgeries were collected from 63 ENT hospitals. Of those, 2,292 involved implanting a CI (2,176 first-time or primary implantations, 99 explantations with immediate re-implantations, and 17 re-implantations following an earlier explantation), while 4 surgeries were explantations only (without re-implantation during the same surgery). Of the 2,108 patients who received a CI in 2022, 1,807 were adults (≥ 18 years, 85.7%) and 301 were children (“pediatric patients”, < 18 years, 14.3%). The largest group of patients treated was aged between 65 and 85 years (*N* = 718, 34.1%), followed by the age group between 45 and 65 years (*N* = 709, 33.6%). Patients older than 85 years made up 2.6% (*N* = 55), patients younger than 1 year 1.9% (*N* = 40), and younger than 2 years 4.9% (*N* = 103). Within the group of pediatric patients (< 18 years, 301 patients), the age group up to 2 years represented just over a third of the pediatric patients treated (34.2%), while the age group under 1 year accounted for 20.9% of the pediatric patients (Fig. 1). There were 1,048 female (49.7%) and 1,060 male (50.3%) patients who received a CI. No patient was documented as diverse.

**Fig. 2:**
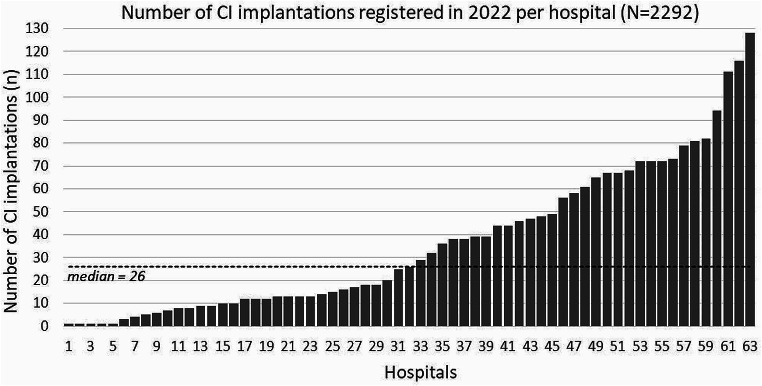
Distribution of the number of cochlear implant (CI) surgeries registered in 2022 among the participating hospitals, dotted line: median value across all 63 hospitals

The number of surgeries registered by a hospital varied between 1 surgery (5 hospitals) up to 128 surgeries (1 hospital) with an average of 36.4 surgeries per hospital (median 26, Fig. 2). There were three institutions that contributed more than 100 surgeries to the registry and 18 hospitals which recorded more than 50 surgeries.

### Unilateral and bilateral implantations

These 2,292 implantations were carried out on a total of 2,108 patients, of whom 113 (5.4%) received a CI in each ear on the same day (simultaneous bilateral implantation). In comparison, a total of 428 patients received a second CI for the first time in their opposite ear, albeit not on the same day (sequential bilateral implantation) with 50 patients having received both their CIs sequentially in 2022. Twenty-one implantations were repeated (multiple) implantations in the same ear. Of the 226 simultaneous bilateral implantations, 162 (71.7%) were performed in children (< 18 years, 81 patients) and 64 (28.3%) in adults (≥ 18 years, 32 patients).

### First language of patients

Data on the main first language was collected for 1,831 patients. German was reported as the first language in 1,586 patients (86.6%). Of the 1,831 patients, 1,568 patients (85.6%) were older than 18 years. In 70% (*N* = 184) of the 263 pediatric patients stated German as their first language. In the group of the adult patients 1,402 patients (89.4%) used German as a first language.

### Onset and duration of hearing loss

**Fig. 3:**
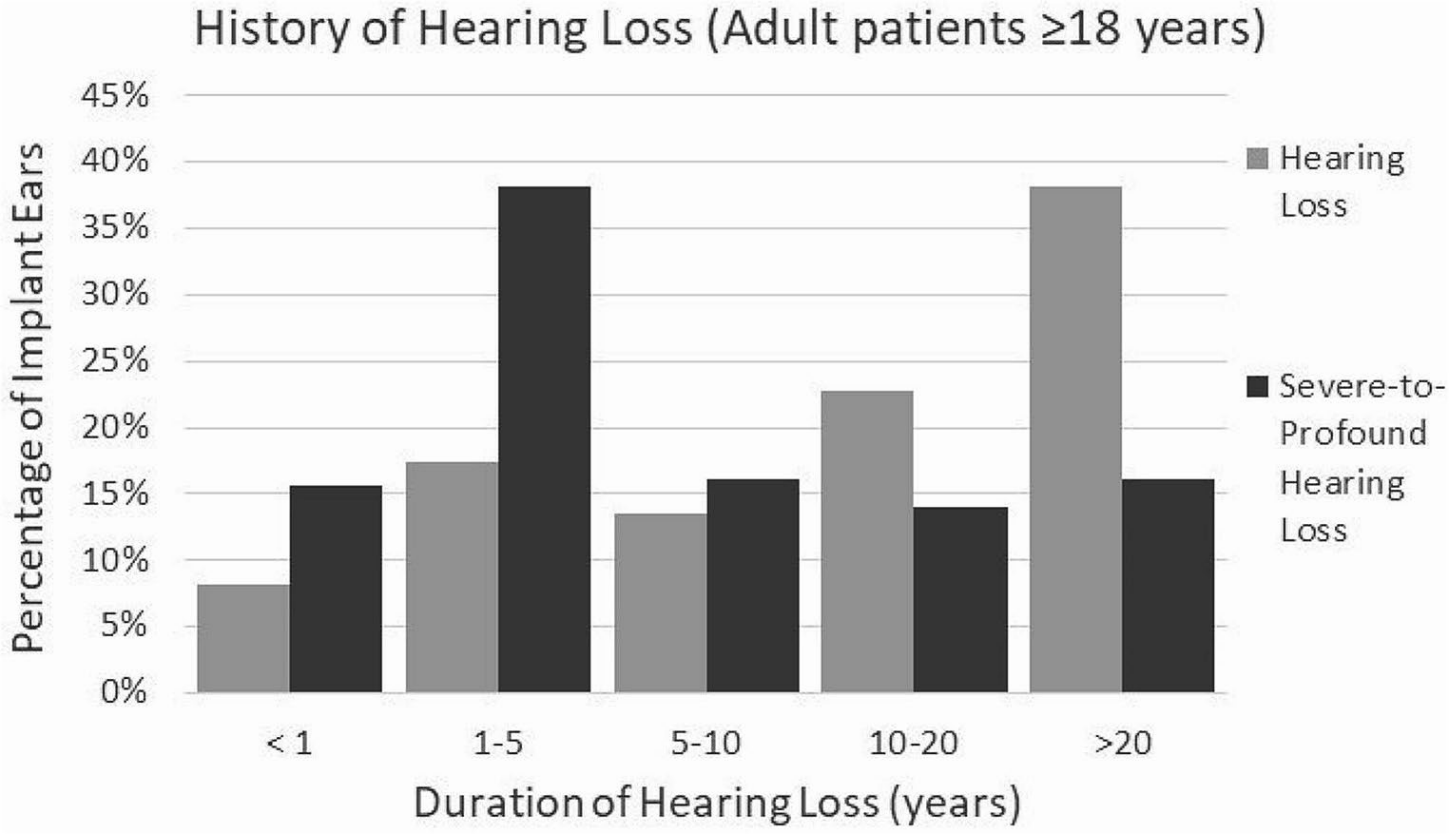
Distribution of hearing loss history among implanted ears for adult (aged ≥ 18 years) patients. In light gray the history of hearing loss (of any severity level) and in dark gray the history of severe-to-profound hearing loss

For the group of adult patients, the onset of hearing loss was documented for 1,578 implantations. In more than half of these implantations (*N* = 960; 60.8%), the onset of hearing loss had already been noticed > 10 years ago, while 129 implantations (8.2%) were reported to have a hearing loss less than 1 year before receiving the CI (Fig. 3).

In addition, the duration of severe hearing loss or deafness in the ear to be treated was recorded. Of the 1,481 recorded ears in adult patients, 231 ears (15.6%) had severe hearing loss or deafness for less than one year. In contrast, 207 ears (14%) had severe hearing loss or deafness between 10 and 20 years, and 238 ears (16.1%) for more than 20 years. The group with hearing loss between one and 10 years included 805 ears (54.3%) (Fig. 3).

It was possible to classify the time of onset of hearing loss in relation to language acquisition for 2,068 implanted ears. The collection of this parameter is particularly relevant for the group of adult patients, for which data were available for 1,739 implanted ears. Of these, 95 (5.5%) showed a prelingual onset of hearing loss and 85 (4.9%) showed a perilingual onset of hearing loss. For the remaining 1,559 (89.6%), a postlingual onset of hearing loss was documented.

### Etiology of hearing loss

The etiology of hearing loss was documented as known or unknown for 1,669 implanted ears. More than half of these (995; 59.6%) had an unknown etiology. Of the remaining 674 implanted ears, specific etiologies were documented in 646 instances. Of these 646 instances, the three most common causes that accounted for 55.4% of the known etiologies, were “age-related hearing loss”, a “genetic cause” and an “infection”. For further details, see Table 1.

**Table 1:** Etiologies of hearing loss*

	Absolute (*n*)	Proportion (%)
Etiology of hearing loss	**1669**	**100%**
Known	674	40.4%
Unknown	995	59.6%
**Documented etiologies**	**646**	**100%**
Age related hearing loss	137	21.2%
Genetic	111	17.2%
Infection	110	17.0%
Meniere’s disease	78	12.1%
Traumatic	63	9.8%
Syndromic	46	7.1%
Ototoxic	37	5.7%
Vestibular schwannoma	32	5.0%
Inner-ear malformation	27	4.2%
Auditory nerve agenesis or abnormality	3	0.5%
Malformation of central hearing pathways	2	0.3%

*Documented only for a part of the cases

### Preoperative hearing aid use

Data were collected from 2,202 implantations regarding the preoperative use of a hearing aid in the ear to be implanted just prior to cochlear implant surgery. For pediatric implantations, it was found that 220 of 387 cases (56.8%) had a hearing aid fitted on the implanted ear, while 167 cases (43.2%) did not. For adult patients, 1,034 out of 1,815 implanted ears (57%) had a hearing aid fitting just prior to implantation, while 781 ears (43%) did not.

### Hearing in the contralateral ear

Information on the hearing ability of the contralateral ear was documented for 2,162 implantations. Hearing loss was found in 1,750 ears (80.9%), whereas normal hearing in the contralateral ear was documented in 412 cases (19.1%, single sided deafness). Among the 412 implantations with contralateral normal hearing, 43 (10.4%) were children and 369 (89.6%) adults.

Data regarding the type of treatment of the contralateral ear at the time of surgery were available for 1,857 implantations. The most common treatment for the contralateral ear was a hearing aid (*N* = 1,064; 57.3%), followed by CI (*N* = 436, 23.5%). Seven cases (0.4%) were fitted with a bone conduction hearing system. No treatment with a hearing system was reported for 343 cases (18.4%). The information “unknown” or “other” was given in 7 cases (0.4%).

### Surgical procedures

A total of 2,296 surgical procedures were documented. These consisted of 2,176 primary CI implantations (94.8%), 99 (4.3%) explantations with immediate re-implantation, 17 (0.7%) re-implantations following previous explantation, and 4 (0.2%) CI explantations without re-implantation. Thus, 2,292 CI implantations were documented in the DCIR in 2022.

### Implants and implantation procedure

In 2022, data on four different manufacturers of CIs and their products was collected for 2,288 implantations (in alphabetical order: Advanced Bionics AG, Cochlear Corp., MED-EL Elektromedizinische Geräte GmbH, Oticon Medical GmbH). Of the documented implants, 1,617 (70.7%) had a so-called “lateral wall” electrode carrier, while 671 implants (29.3%) had a “preformed” electrode carrier.

For 2,284 implantations, information on the surgical insertion of the electrode could be obtained. In 1,911 cases (83.6%), the electrode was inserted via the round window. In 241 cases (10.6%), the electrode was inserted via a cochleostomy (other: 132; 5.8%).

For 2,265 implantations, data on the insertion depth was collected. Complete insertion was documented for 2,221 cases (98.1%), while in 44 cases (1.9%) there was partial insertion of the electrode.

Data on intraoperative measurement (e.g. stapedius reflex, neural response telemetry, eCAP) was collected for 2,241 implantations. In 2,082 cases (92.9%), the results were rated “normal”. In 134 cases (6%), the results were not “normal”. In 25 cases (1.1%), no intraoperative recording of electrically evoked compound action potential (eCAP) was done.

### Radiological position control of the electrode carrier

Data on radiological position control of the electrode carrier were available for 2,176 (94.9%) implantations. Of these 2,176 implantations, information on the time of the examination was provided in 2,046 cases. In most cases, the radiological position control was performed postoperatively (1,635 of 2,046 implantations, 79.9%), and in 411 (20.1%), it was performed intraoperatively. Of the above 2,176 implantations where a radiological position control was performed, 2,001 implantations were documented as having either a regular (*N* = 1,965, 98.2%) or an irregular (*N* = 36, 1.8%) position of the electrode carrier. The used radiologic method was documented for 2,086 out of 2,294 implantations. The following methods were used: CT in 839 implantations (40.2%), DVT in 433 implantations (20.8%), conventional X-ray in 772 implantations (37.0%), other in 42 implantations (2.0%).

### Explantations

In 2022, 103 surgeries were documented in which a CI had to be explanted. Of these, 99 cases (4.3% of all 2,296 surgeries) were explanted and immediately re-implanted, and 4 cases (0.2%) of CI explantation without re-implantation. Within the registry, the causes of explantation were assigned based on the recommendation of the 2005 “European Consensus Statement on Cochlear Implant Failures and Explantation” for categorization [19] (Table 2). The most common cause documented in the survey was class B2 (“Performance Decrement”) with 43 cases (41.7%), while a “Medical Reason” (e.g. explantation due to infection) was documented in 14 cases (13.6%).

**Table 2:** Reasons for explantation

Failure Category	Guideline Class	Absolute (*n*)	Proportion (%)
Total		103	100%
Functional device	A1	7	6,8%
Soft failure: functional device with decreased performance	A2	5	4,9%
Technical characteristic decrement	B1	4	3,9%
Performance decrement	B2	43	41,7%
Hard device failure	C	20	19,4%
Medical reason	D	14	13,6%
No data provided	-	10	9.7%

### Postoperative fitting of the audio processor

Data from 1,371 patients or 1,549 CI-fitted ears were collected to record the postoperative fitting of the audio processor. For 440 ears (28.4%), it was documented that a “single-unit processor” was used. A behind-the-ear system was used in 1,034 ears (66.8%). In 75 cases (4.8%), the unspecific information “other” was given, so that no clear assignment of the processor type used was possible for this subgroup.

### Postoperative CI use

Data on the usage time of CI use was collected for 1,060 patients (1,192 CI-fitted ears). Data logging was used for 963 ears (80.8%) and for 186 ears (15.6%) information was provided by the patient or their parents. “No information” was indicated for 43 ears (3.6%). The average daily usage time of the CI for each ear was 11.0 h.

### Complications

Complications were recorded under the categories “hospitalization due to Cl-related complications”, “incorrect position of the electrode carrier requiring revision”, “meningitis after CI fitting”, “death in connection with CI fitting”, or postoperative facial nerve dysfunction (House Brackman grades I to VI). As more than one category may be present per reported case, the summary of these complications is shown in Table 3. Please note that in 3 cases, some of the categories were left blank. These are interpreted as being “No complication”.

**Table 3:** Complications

Case	Hospitalization due to Cl-related complications	Incorrect position of the electrode carrier requiring revision	Meningitis after CI fitting	Death in connection with CI fitting	Postoperative facial nerve palsy House Brackman Grades I-VI
1	Yes	No	No	No	Grade I (Normal/none)
2	Yes	No	No	No	Grade I (Normal/none)
3	Yes	No	No	No	Grade I (Normal/none)
4	Yes	No	No	No	Grade I (Normal/none)
5	Yes	No	No	No	Grade I (Normal/none)
6	Yes	No	No	No	Grade I (Normal/none)
7	Yes	No	No	No	Grade I (Normal/none)
8	Yes	No	No	No	Grade I (Normal/none)
9	Yes	No	No	No	Grade III (Moderate)
10	Yes	Yes	No	No	Grade I (Normal/none)
11	No	No	No	No	Grade II (Mild)
12	No	No	No	No	Grade II (Mild)
13	No	No	No	No	Grade IV (Moderate severe)
14	No	Yes	No	No	Grade I (Normal/none)
15	No	Yes	No	No	Grade I (Normal/none)
16	No	Yes	No	No	Grade IV (Moderate severe)
17	n/a	Yes	n/a	n/a	Grade I (Normal/none)
18	n/a	n/a	n/a	n/a	Grade II (Mild)
19	n/a	n/a	n/a	n/a	Grade II (Mild)

In total, 19 complications were recorded, with 10 of these requiring “hospitalization due to Cl-related complications”, while the remaining 9 cases did not. Of these 10 “hospitalized” cases, only one was also documented as “incorrect position of the electrode carrier requiring revision”.

Of the 9 cases which did not require hospitalization, 4 cases were indicated as involving “incorrect position of the electrode carrier requiring revision”. Altogether, a total of 5 cases involved an “incorrect position of the electrode carrier requiring revision”, with only one of these 5 cases requiring hospitalization. There were no cases of documented “meningitis after CI fitting" or “death in connection with CI fitting”.

Postoperative impairment of facial nerve function of varying degrees of severity (from mild to moderate severe dysfunction) was reported in 7 of the 19 cases with complications. Four of these were mild (House-Brackman grade II), one was moderate (grade III) and two were moderate severe (grade IV). One patient with a grade III facial nerve dysfunction involved hospitalization, while one patient with a grade IV was also indicated as involving an “incorrect position of the electrode carrier requiring revision”. No case of a severe or total dysfunction (House-Brackmann grade V or VI) was documented.

## Discussion

After several years of preparation and despite organizational, technical, legal, and financial challenges, the German Cochlear Implant Registry (DCIR) began enrolling patients in January 2022. Over the course of the first 12 months, data from 2,292 implantations for 2,108 patients from 63 hospitals were transferred to the registry. The present study provides an initial insight into the experiences gained and the data collected within 2022. Due to the considerable amount of data, the focus of this paper is on presenting demographic results only. Audiological outcome data will be analyzed and presented separately.

In the first year of the registry’s operation, 75 hospitals had contractually agreed to participate in the DCIR. Based on the estimation that CI care is currently offered by around 100 hospitals in Germany [11], the number of hospitals that had declared their willingness to participate in the registry is promisingly high. In addition, all federal states and all large hospitals in Germany are represented in the registry at present. As participation in the DCIR is not only voluntary, but also involves direct and indirect costs for participating institutions [7], 75 hospitals within the first year can be considered very promising, demonstrating the high motivation and great support of the DCIR by ENT physicians and hospitals in Germany.

Only 63 of the mentioned 75 hospitals submitted data to the registry in 2022. Despite the willingness of additional 12 hospitals to contribute to the DCIR, there were organizational, technical, or legal challenges that prevented the submission of data. Various reasons for this were mentioned, such as appointing or training responsible staff, required time or technical effort, but also the fact that relevant data to the registry may not have been collected. In some cases, the type of data transfer preferred by the participating hospital caused a delay that made active participation in the registry difficult. However, it is encouraging that almost 85% of the participating hospitals (63 of 75) were able to contribute data to the registry already during the first year of operation.

It is not yet possible to draw final conclusions on the exact number of implantations performed in Germany from those that are documented in the DCIR. Various reference data can be used to estimate the total number of procedures in 2022. The most reliable information currently available is data from the German Federal Statistical Office. Comparative figures are available for 2022 [12]. The number of new CI implantations in 2022 was 4,271. There were also 276 CI exchanges (re-implantations). This resulted in a total of 4,547 CI implantations in 2022. Considering this, the number of 2,292 implantations registered in the DCIR in 2022 seems relatively low. Approximately 50% (2,292 of 4,547) of CI implantations were recorded in the first year of the registry’s operation. As the DCIR is currently based on the voluntary participation of hospitals, this is an encouraging result for the first year.

The data transfer to the registry is expected to increase in the future due to the parallel certification of participating hospitals as “certified CI care hospitals”. This certification program has recently been introduced throughout Germany [13]. The active participation of a hospital in the DCIR [7] is an essential prerequisite for the successful acquisition of this certificate. Thus, the close link between registry participation and certification acts as a positive feedback loop to increase the future data quality and quantity of the DCIR.

### Age and gender

Approximately 86% of the registry patients were adults and approximately 14% were children (younger than 18 years). There was no uniform age distribution between children and adults. Children up to 2 years of age accounted for a third (34%) of all children treated. Cochlear implantation within the first year of life accounted for about 21% of all children treated but only 2% of all CI implanted patients. According to the German CI clinical practice guideline [5], CI implantation in children with congenital hearing loss should ideally be performed within the first year of life. A recent study describes a clear difference in speech understanding in children depending on the time of CI treatment [14]. Children implanted in the first year of life showed significantly better results than those implanted in the second year. Documentation of the timing of CI therapy in children based on the DCIR can therefore in the future contribute to ensuring an optimal CI care process for children.

Cochlear implantation in adult patients shows peaks between the ages of 45 and 65 and between 65 and 85. Based on the presented data, the number of implantations in very young and very old patients is similar in Germany. Cochlear implantation results in a significant increase in the quality of life, cognitive abilities and ultimately autonomy, especially for older patients [15–17]. Based on the data collected by the DCIR to date, there appears to be a possible underuse in patients older than 85 years. As the success of CI treatment is undisputed, particularly in older patients, this should provide a strong motivation to increase CI treatment in this patient group.

### Results on the first language

Most patients provided with a CI in 2022 use German as their first language. Only 13% were non-native speakers who are more likely to face challenges regarding language-related integration in addition to pure hearing and speech rehabilitation. Children fitted with a CI require special consideration regarding adequate hearing and speech development. In this group, approximately 30% are not using German as their first language. This should also be considered when structuring the hearing rehabilitation program and allocating the necessary resources to guarantee individualized support for patients.

### Onset of hearing loss

The data analysis shows that most patients suffered hearing loss during or after language acquisition. Prelingually deafened patients make up the minority of the patients treated. It will be interesting to assess the extent to which the hearing and speech test results achieved in this group will change over time. A detailed analysis shows that for most patients, hearing loss began more than ten years ago. This confirms the clinical perception that the predominant proportion of adult patients suffer from progressive hearing loss. However, there is also a relevant number of patients with sudden hearing loss, i.e. onset of hearing loss less than 1 year before implantation).

A considerable number of adult patients reported more than 10 years or even more than 20 years of profound hearing loss or deafness prior to CI surgery, suggesting that several patients could have been treated with a CI long before the treatment actually took place. We expect that continuous DCIR data analysis will provide explanations for this phenomenon.

### Hearing aid use before CI fitting

Almost 60% of the adult patients were using a hearing aid in the ipsilateral ear prior to the CI surgery. No information was given why the remaining 40% did not use a hearing aid. However, since most patients actively used a hearing aid until immediately before cochlear implantation it can be assumed that these patients had residual hearing. This is consistent with the clinical experience that most patients in Germany who receive a CI are not completely deaf. The data collected here is therefore also in line with the current CI guideline which recommends a CI in patients with a word recognition score of ≤ 60% monosyllables in the Freiburg Speech Test at 65 dB sound pressure level (SPL) in quiet [5].

### Hearing ability of the contralateral ear

The study showed considerable differences in the hearing ability of the contralateral ear. This also represents the current indication for CI care in Germany. As each ear is individually considered, any combination (e.g., bilateral profound hearing loss or deafness, moderate contralateral hearing loss, contralateral normal hearing) presents an accepted indication for CI treatment. Especially many patients with normal contralateral hearing (“single-sided deafness”, SSD), are treated with a CI in Germany. The analysis of the data collected here showed a proportion of approximately 20% SSD patients. It will be of particular interest in the future to see how the speech understanding achieved in this group will differ from that of other patient groups.

### Etiology of hearing loss

Considering the cases documented with a known etiology (approximately 40%), it must be critically questioned whether an objective verification of the reported causes would be possible. Mostly, a history-based cause for the hearing loss was given without any objective evidence. These findings reveal a persistent dilemma in clinical otology, since for many patients there is no causal explanation for their sensorineural hearing loss.

### Surgery

All four manufacturers represented in Germany in 2022 were also represented in the registry. In the predominant number of cases (70%), “lateral wall electrodes” were used and in most cases, the electrode was inserted via the round window (84%). Even though there was variation both in terms of the use of the implant and the use of the respective electrode carrier, the relatively low use of preformed electrodes (30%) is noteworthy. Future analyses will provide an insight into the reasons for the decision in favor of a specific implant or the use of a specific electrode array.

Complete insertion of the electrode carrier was achieved in almost all (98%) cases. The intraoperative measurements (e.g. stapedius reflex, neural response telemetry, eCAP) showed “regular” results in 93%. Intraoperative measurement was not performed in 1% of all implantations although this is clearly recommended in the current guideline. Analyzing the reasons for not carrying out the intraoperative tests as well as analyzing long-term hearing results in cases of irregular intraoperative test results will be of interest in the future. In the long term, this may also provide an answer to the question of the relevance of intraoperative functional measurements for the success of the treatment.

### Radiological position control of the electrode carrier

In most cochlear implantations, the position of the electrode carrier was confirmed radiologically. This was predominantly carried out postoperatively, but in almost a quarter of all cases intraoperatively. Radiological position control was only omitted in a small proportion of the patients treated (5%). No information on the reasons for this can currently be derived from the registry. In the future, it will be interesting to record whether the number of radiological exams changes, as some studies propagate electrophysiological measurement methods that could possibly serve as a substitute [18]. If such approaches prove successful, this could also be detectable in future registry data. An “irregular position” of the electrode carrier was documented in approximately 2% of the cases. Therefore, radiological position control of the electrode carrier seems to make sense at present.

### Explantations

There were 103 CI explantations (4.5%) among the total of 2,296 surgical procedures documented in 2022. The most common cause was technical failure which can be considered as a malfunction or complete failure of the implant. A study based on the “European Consensus Statement on Cochlear Implant Failures and Explantation” [19] shows that “Performance Decrement” (B2) is the most common cause of explantation. Classification of the 103 cases of CI explantation in 2022 is currently only possible to a limited extent as the number of CI surgeries documented in the registry is estimated to only represent approximately 50% of the implantations performed in Germany in 2022. With an increasing database, the DCIR will make an important contribution to the issue of quality control and implant safety in the future.

### Post-operative fitting of the audio processor

The DCIR also contains data on the technical details of hearing rehabilitation, e.g., the type of audio processor (design). An example of this is the use of Behind-The-Ear (BTE) system versus Single-Unit-Processors (SUP). In 2022, most patients chose a BTE system and only around 30% a SUP. It will be of interest in the future, whether the use of a specific processor and the CI system will influence the audiological outcomes.

Data on daily use can already be collected from the registry at this stage, with an average of 11.0 h per implant. It is worth noting that in the majority of cases, data from so-called “data logging”, i.e., information objectively read from the processor software, has already been included in the registry. This also opens up interesting possibilities for assessing patients’ hearing performance in the future.

### Complications

In 2022, data on complications was recorded in 19 (0.8%) of 2,292 implantations. The hospitals also actively documented if there were no complications. The data also showed a very low rate of serious complications and an absence of cochlear implantation related severe or complete facial nerve dysfunction, meningitis or death as a result of CI surgery. Future studies will also allow the analysis of potential long-term complications or their changes of time.

### Limitations

For some of the parameters collected, it was not possible to analyze a complete data set for all documented CI procedures in the first year. A critical analysis of the data quality generated in the first phase of the DCIR therefore indicates a need to improve “mandatory documentation” for certain parameters. The current study therefore has also made an important contribution to further improving the data quality of the DCIR. Changes to the use of the registry in this respect have already been decided upon and are currently being implemented (e.g. mandatory documentation of complications). In the long term, the recording of possible complications of CI therapy may also be of great importance for manufacturers of implant systems in order to meet the requirements of the European Medical Device Regulation (MDR) [20].

Another current limitation of the DCIR is the incomplete data collection from individual hospitals. In 2022, the number of records per hospital varied between 1 and > 100. This currently results in incomplete documentation but may also result in an over- or under-representation of individual hospitals and their specific results in the registry. The long-term aim is to achieve complete data collection from all hospitals. However, as this is currently voluntary for both hospitals and patients, this is an ongoing challenge, because at present data can only be added to the registry, if a patient and the hospital give consent. This may be particularly relevant for the approximately 25 hospitals in Germany that are not yet participating in the DCIR. These are likely to be smaller institutions. The extent to which there are differences in the quality of CI care provided by these facilities can only be speculated at present. To solve this problem, political decisions have been taken in Germany to make the recording of implant data mandatory for all hospitals and patients in the future. The basic legal framework has already been created including CI therapy [21]. Therefore, it is clearly foreseeable that a future mandatory CI registry will also close any potential data gap.

## Conclusion

In retrospect, several organizational, legal and technical challenges had to be overcome during the first year of operation of the DCIR. Although technical improvements are ongoing, the registry and the data presented can already contribute to the understanding of the CI care in Germany. Fortunately, this work shows that the DCIR contains data on all relevant patient groups, both in terms of age (< 1 year to > 85 years), gender distribution, type of treatment (e.g. unilateral, bilateral), short-term (< 1 year) or long-term (> 20 years) deafness as well as single-sided deafness. The same applies to patients with residual hearing (electroacoustic stimulation, EAS, hybrid). The cases documented in the DCIR therefore represent the full range of treatment options with a CI. This, together with lifelong follow-up, will provide a very useful database for the long-term evaluation of CI outcomes, the underlying treatment process, possible complications and the safety of the implants. Although the DCIR is still in its early stage, it is already providing interesting insights and therefore is an important source of data sets for the development of scientifically based treatment guidelines. Future data analysis will document changes in indications, outcomes, and care processes in a standardized and nationwide manner, particularly over the next years. The “real world data” collected in the DCIR can thus make a valuable contribution to the quality control of CI care in Germany.

With the registry initiative, the DGHNO-KHC has made a relevant contribution to CI care in Germany. The concept of a national CI registry [7] and the associated certification of CI hospitals [13, 22] could perhaps serve as an example for other countries. In the long term, an internationally standardized CI registry should also be discussed to collect data on the quality of CI care worldwide. Patients worldwide could then benefit from this to identify best-practice care processes for cochlear implant therapy based on a scientific benchmark.
